# Impact of long-term loquat cultivation on rhizosphere soil characteristics and AMF community structure: implications for fertilizer management

**DOI:** 10.3389/fpls.2025.1549384

**Published:** 2025-03-13

**Authors:** Yu Zhang, Zhenteng Liang, Liangxun Zheng, Xinyang Wang, Hui Chen, Tingying Xu, Ming Tang

**Affiliations:** ^1^ State Key Laboratory of Conservation and Utilization of Subtropical Agro-Bioresources, College of Forestry and Landscape Architecture, South China Agricultural University, Guangzhou, China; ^2^ Boone Pickens School of Geology, Oklahoma State University, Stillwater, OK, United States

**Keywords:** loquat varieties, arbuscular mycorrhizal fungi, AMF diversity, nutrients uptake, soil function

## Abstract

The role of arbuscular mycorrhizal fungi (AMF) in assisting the growth of different fruit tree species is well-established, yet the impact of loquat cultivation under long-term human management on the rhizosphere soil characteristics and AMF community structure remains unresolved. To address this knowledge gap, we collected roots and soil samples from 20-year-old loquat in a loquat germplasm resources nursery with consistent water and nutrient conditions including one wild species (YS), three pure species (GXQH, MHH, DWX), and four hybrid species (ZJ90, JT, JTH, ZU7). Our analysis revealed that AMF colonization rates ranged from 40.57% to 65.54%, with *Glomus* (30.72%) and *Paraglomus* (29.46%) being the dominant genera across all varieties. *Paraglomus* dominated in pure species, while *Glomus* prevailed in wild species. YS exhibited the highest AMF richness than cultivars. Significant variations in soil nutrients and enzyme activities in the rhizosphere among different varieties. Total nitrogen (TN) and total potassium (TK) were significantly negatively correlated with relative abundance of AMF genera, suggesting that nitrogen and potassium may reduce AMF abundance. Mantel test showed that total carbon (TC) and soil organic matter (SOM) were the key factors influencing AMF community composition (*P*<0.01). These nutrients were positively correlated with dominant AMF genus (0.06, R^2^ = 0.05) but negatively with rare genus such as *Ambispora* (−0.08, R^2^ = 0.24). Overall, these findings confirmed that plant varieties or genotypes drive changes in AMF communities and further demonstrated that long-term nutrient enrichment reduces the diversity of loquat rhizosphere AMF communities. These results support the use of AMF biofertilizers and reducing fertilizer application.

## 1 Introduction

Germplasm repositories are essential for ensuring future food security. However, they are only viewed as storage facilities for resource conservation, leading to a lack of attention to the relationship between soil microbial ecosystems and germplasm species (Wang et al., 2024). Arbuscular mycorrhizal fungi (AMF), as a crucial group of soil fungi, can form mutualistic symbiosis with most vascular plants, playing significant roles in material exchange and information transfer between soil and plants (Smith and Read, 2008; Maillet et al., 2011; Liu et al., 2011). This symbiotic relationship entails AMF promoting host plant growth, enhancing water and mineral nutrient absorption, and increasing resistance to biotic and abiotic stresses (Zhu et al., 2022; Wang and Rengel, 2024; Wu et al., 2024a). In agriculture, AMF primarily colonizes plant roots, utilizing their mycelium to provide nutrients such as phosphorus and nitrogen to host plants, thereby enhancing productivity (Wu et al., 2024b; Sha et al., 2019). The extent of AMF colonization in various plant roots and the diversity of AMF in rhizosphere soil are interconnected with nutrient levels and soil characteristics (Sheldrake et al., 2018). Previous research indicated that the composition and distribution of AMF communities in ecosystems are strongly influenced by environment such as geographical features and plant types (Chen et al., 2017; Mhlanga et al., 2022). Many diversity studies endeavors were conducted under field conditions, where alterations in soil physical and chemical properties played a crucial role. In subtropical regions, soils typically exhibit low phosphorus levels, leading to the widespread assumption that the richness and diversity of AMF communities are restricted (Wen et al., 2024). However, recent studies, leveraging high-throughput sequencing technologies, suggested that both the abundance and diversity of AMF in such environments had been significantly underestimated (Luo et al., 2020; Kajihara et al., 2022). Therefore, understanding the biogeographical patterns and the drivers behind them is essential for preserving ecosystems, the specificity of AMF in germplasm resource banks needs to be studied (Chen et al., 2023; Zhang et al., 2024b).

Loquat is well-known for its high yield and economic significance in southern China. Its fruit is nutritious and delicious (Jing et al., 2023), and its leaves and flowers contain a variety of medicinal ingredients (He et al., 2020). Loquat seed resource garden located in Guangzhou, China, contains more than 300 germplasm resources, representing 30 loquat varieties, Therefore, this GenBank provides a promising opportunity to explore AMF colonization parameters and community structure. However, loquat cultivation faces challenges due to its shallow and underdeveloped root system, compounded typically by phosphorus-deficient soils (Xu et al., 2020), limiting its growth and nutrient accumulation. While using wild loquat germplasm as rootstock has improved the survival rate of seedlings when breeding loquat varieties, it has not fundamentally solved the problem of the shallow root system and susceptibility to lodging. Moreover, some germplasm resources are limited by these root system challenges and cannot be inherited effectively (Xu et al., 2020). Therefore, it is particularly important to protect loquat germplasm using biological methods and explore the community distribution of AMF in different loquat germplasms. Analyzing the composition and changes of AMF communities between wild germplasm and cultivated germplasm will not only help to use AMF to improve the stress resistance of loquat, but more importantly, clarify the important role of symbiotic relationships in germplasm resource protection. Establishing symbiotic bonds between fruit trees and AMF relies on the presence of AMF spores in rhizosphere soil (Wahab et al., 2023). Certain species of AMF have been identified to stimulate the growth of loquat roots, increasing their dependence on mycorrhization and facilitating soil nutrient uptake (Zhang et al., 2014), with notable species including *Acaulospora laevis*, *Funneliformis caledonium*, and *Funneliformis mosseae* (Wang et al., 2007). Current strategies to maintain production and increase income primarily focused on improving planting density to promote root growth and fertilization to enhance nutrient absorption (Polat et al., 2005; Huang et al., 2021). Excessive nutrient inputs, however, can lead to severe microbial stoichiometric imbalances, forcing microbes to mobilize scarce elements by producing specific extracellular enzymes to maintain their stoichiometric balance (Xiang et al., 2024). Therefore, it is imperative to investigate the dynamics of AMF communities after artificial nutrient supplementation, as this holds significant significance for the protection of germplasm resources.

To test our hypothesis, we conducted a comprehensive study within the loquat seed resource garden, collecting fine root and rhizosphere soil samples from eight different loquat fruit trees. Our study included quantifying mycorrhizal colonization in fine roots, analyzing the physicochemical properties and spore density of AMF in the rhizosphere soil, and using amplicon sequencing to assess changes in AMF community composition. Additionally, we systematically evaluated the direct and indirect effects of loquat varieties on AMF colonization and community structure using structural equation modeling (SEM). This study aims to achieve three main objectives: (1) Are there differences in AMF species between wild and cultivated loquats under identical water and fertilizer conditions? (2) Are these differences caused by the plant’s genetic traits or by human intervention in the environment? (3) What role can these differences play in the conservation of loquat germplasm resources? This study provides some progress in our understanding of community changes in AMF and utilization of AMF for the conservation of loquat germplasm resources in the future.

## 2 Materials and methods

### 2.1 Sample site

The experimental field is located at the loquat germplasm resource nursery in Guangzhou, Guangdong Province, China (23°9′27″N, 113°21′11″E). This region experiences a subtropical monsoon marine climate typical of South Asia, with an annual average temperature ranging from 21.8°C to 22.9°C and an average annual rainfall between 1384.4 mm and 2278.3 mm. The soil type at this site is classified as latosol. The nursery serves as a long-term experimental platform for loquat variety improvement, consisting of around 30 varieties (approximately 300 trees). Annual management practices include regular weeding and irrigation, performed as required based on actual situation. Fertilization is conducted two to three times per year. Foliar fertilizers are introduced during the flowering spike stage. While fruit-strengthening fertilizers are applied as fruiting progresses. Flowers and fruits are then thinned according to their abundance. After harvest, peanut bran is mainly applied. All varieties are maintained on this land with consistent initial soil nutrient levels and fertilizer input conditions.

### 2.2 Soil sample collection

Soil samples were collected from the rhizosphere of eight loquat cultivars, all with a consistent growth history of approximately 20 years. Four of the most valuable hybrid varieties (JT, JTH, ZJ90, ZU7) and three pure varieties (DWX, MHH, GXQH) were selected from the germplasm resource bank for study, while a wild type (YS) under the same conditions was used as a control (**Supplementary Table S1**). The experimental sampling was conducted in October 2022, before the loquat fruiting period, when the soil was relatively stable and free from human interference. Prior to sampling, about 3 cm of topsoil and debris were removed. Roots and soil samples were collected at a depth ranging from 5 cm to 30 cm and at a distance greater than 50 cm from the trunk, with soil attached to the fine roots shaken off. Samples were collected from three directions around the loquat, thoroughly mixed into one composite sample, and each variety was sampled at least 3 times. In total, 24 soil samples were obtained and analyzed for physicochemical properties. Each sample was divided into two parts: one part was stored at −80°C for AMF sequencing, while the other part was air-dried for analysis of soil properties, enzyme activity, and AMF spore density.

### 2.3 Root colonization and AMF spore

Fresh rootlets were collected, cut into approximately 1 cm to 2 cm in length, immersed in FAA fixative solution, and stained using the method described by Phillips and Hayman (1970) to determine the mycorrhizal colonization rate. To quantify the percentage of root colonized by AMF, a modified line intersection method described by McGonigle et al. (1990) was employed. This method involved randomly selecting 100 lines intersections within each root sample and examining them for the presence of hyphae, vesicles, and arbuscules, providing an estimation of AMF colonization in the roots.

According to the approach by Brundrett et al. (1994), the wet screen decantation sucrose gradient centrifugation method was utilized to separate spores from the soil surrounding loquat roots. Subsequently, these spores underwent microscopic examination for the observation and recording of various morphological characteristics. Spore size measurements were conducted using ImageJ (the National Institutes of Health in Bethesda, MD, USA). We referred to the International Bacterial Collection Center (http://invam.ku.edu) and named the fungal species following the fungal species list of the arbuscular mycorrhizal classification system (Wang et al., 2007).

### 2.4 Soil sample analysis

The pH was determined using an electronic pH-meter at a soil to water ratio of 1:5. Soil organic matter (SOM) content was measured using the external potassium dichromate heating method. Total carbon (TC) and total nitrogen (TN) in the soil were determined through an Elementar C/N Analyzer (Eltra, Haan, Germany). Nitrate (NO_3_-N) and ammonium (NH_4_^+^-N) nitrogen were determined using the KCl solution extraction method (Keeney and Nelson, 1982). Total phosphorus (TP) and available phosphorus (AP) were extracted using the NaOH melting method and NaHCO_3_ solution extraction method, respectively, and determined by the molybdenum antimony colorimetric method (Olsen and Sommers, 1982). Total potassium (TK) and available potassium (AK) were extracted using the HF-HClO_4_ digestion method and NH_4_OAc solution extraction method (Bao, 2000), respectively, and determined using an atomic absorption spectrophotometer. Soil enzyme activity was determined following the method described by Guan (1986).

### 2.5 Soil DNA extraction and high-throughput sequencing

The Illumina MiSeq high-throughput sequencing technology (Shanghai Personal Biotechnology Co., Ltd., China) was employed to conduct ASV clustering analysis, species annotation, and database comparison, for investigating the variation characteristics of the AMF community composition and diversity in the rhizosphere soil of different loquat cultivars. Each species was measured at 10 individuals. The inferential scale in this example was at the species level, with the factor of interest being the variety, and variations observed at the species level. Each level of the factor was represented by three appropriate replication units.

To further analyze the presence of AMF in the soil, soil genomic DNA extraction kits were used to extract AMF DNA from the soil samples. For the initial PCR amplification, universal primers targeting the fungal 18S rDNA region, specifically Geo-F (5’-CAGTAGTCATATGCTTGTCTC-3’) and Geo-R (5’-ACCTTGTTACGACCTTACTTTCC-3’), were utilized. For the second PCR amplification, primers AML-F (5’-ATCAACTTTCGATGGTAGGATAGAGA-3’) and AML-R (5’-GAACCCAAACACTTTGGTTCC-3’) were employed to target the AML-F/AML-R region within the ribosome 18S rDNA, resulting in an amplification fragment of approximately 800 base pairs in length.

### 2.6 Impact of loquat varieties on AMF community and structure

To assess the α-diversity and β-diversity of AMF, community RNA fragments were sequenced using the Illumina platform. A normalized ASV table was employed to assess variations in microbial community composition. We set the minimum rarefaction depth to 10 and selected 10 evenly spaced depth values between the minimum depth and 95% of the lowest sequencing depth in all samples. At each depth value, the table will be rarefied 10 times, and the selected alpha diversity metrics will be calculated. The mean score at the maximum rarefaction depth will be used as the alpha diversity index. Subsequently, a Bray-Curtis distance matrix was computed from the normalized data, and PCoA and NMDS analyses were performed on the distance matrix. With the aid of R script, PCoA and NMDS coordinates were obtained for each sample point and plotted on a two-dimensional scatter plot to visually represent the differences in community composition. The induced_subgraph function of graph was utilized to extract the nodes with the top 100 average abundance (default) according to the abundance of nodes (ASV) and build a dominant species subnetwork, followed by visualization using the graph package for visualization.

### 2.7 Statistical analyses

Statistical analysis was conducted using Microsoft Excel and SPSS26.0 (SPSS Inc., Armonk, NY, USA). Variance analysis and significance testing (*P*<0.05) were performed using the Duncan test. Figures were generated using Origin 2023 (Electronic Arts Inc., DE, USA) and the free online platform Personalbio GenesCloud (http://www.genescloud.cn). Correlation analysis was conducted using R Studio (version R 4.2.2; Foundation, New Zealand), and pathway analysis was constructed using SPSS Amos 26.0 (IBM, SPSS).

One-way ANOVA was used to analyze the changes in mycorrhizal colonization rates, soil physicochemical properties, and soil enzyme activities of different loquat varieties. To elucidate the relationship between loquat, soil, and AMF, a structural equation model was constructed to hypothesize the causal relationships among six variables, including different loquat varieties, soil factors, and AMF parameters. The two soil factors considered were soil physicochemical properties and soil enzyme activity. The AMF parameters included mycorrhizal colonization, spore density, and AMF diversity, which encompassed abundance, diversity, and evolutionary diversity. In the model, the mycorrhizal colonization index included spore colonization rate, arbuscular abundance, and hyphal colonization rate. Additionally, we used the first two axes of principal component analysis (PCA) to quantify the data for each factor. Correlated relationships were represented by arrows (positive correlation indicated by a red arrow, negative correlation indicated by a blue arrow), while independent relationships were indicated by solid or dashed lines based on the significance level (*P*<0.05).

## 3 Results

### 3.1 Soil properties and enzyme activity in loquat rhizosphere

**Figure 1:**
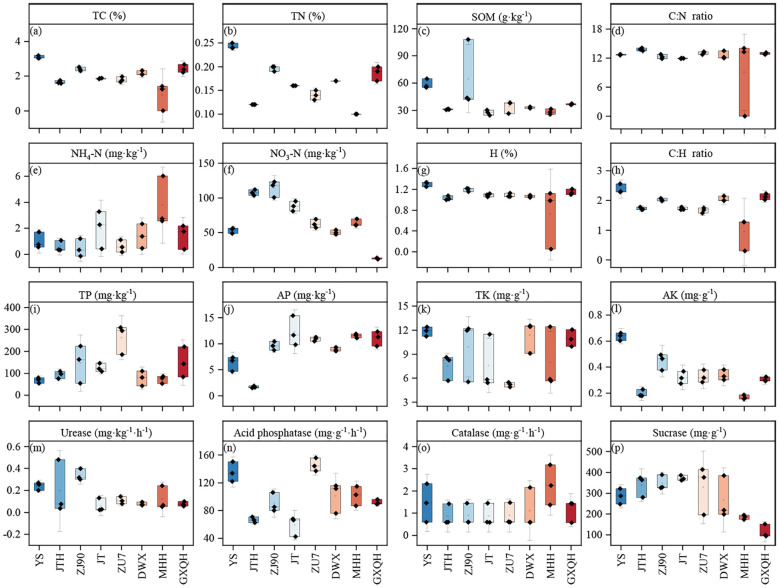
Soil properties and enzyme activities in different varieties of loquat. a-l are soil physical and chemical properties, representing total carbon **(a)**, total nitrogen **(b)**, organic matter **(c)**, carbon-nitrogen ratio **(d)**, ammonium nitrogen **(e)**, nitrate nitrogen **(f)**, hydrogen **(g)**, carbon-hydrogen ratio **(h)**, total phosphorus **(i)**, available phosphorus **(j)**, total potassium **(k)** and available potassium **(j)**. m-p are soil enzymes, representing urease **(m)**, acid phosphatase **(n)**, catalase **(o)** and sucrase **(p)** respectively. TC, total carbon; TN, total nitrogen; SOM, soil organic matter; TP, total phosphorus; AP, available phosphorus; TK, total potassium; AK, available potassium.

Considerable changes in the physicochemical properties and enzyme activities of the rhizosphere soil were observed, with notable differences among various loquat varieties revealed by variance analysis (**Figures 1a–p**). YS exhibited significantly higher TP (**Figure 1i**), AP (**Figure 1j**), and SOM (**Figure 1c**) compared to other species. Conversely, ZJ90, JT, and ZU7 had significantly higher TP, AP and NO_3_-N (**Figure 1f**) than other varieties. DWX, MHH, and GXQH had higher levels of NH_4_^+^-N and TP compared to hybrid varieties. Furthermore, soil enzyme activities varied across loquat varieties. Catalase activity remained relatively stable overall, with no significant differences observed. However, urease activity showed significant variability, with ZJ90 displaying the maximum root urease activity of 0.34 mg/kg, while JT exhibited the minimum at only 0.06 mg/kg. Acid phosphatase activities were notably higher in YS and ZU7 compared to JTH and JT, with ZU7 showing 1.47 times higher activity than JT. Sucrase activity did not differ significantly between YS and hybrids but was significantly higher than that of pure varieties. For instance, JT exhibited sucrase activity 2.24 times higher than GXQH.

### 3.2 AMF colonization in loquat rhizosphere

**Figure 2:**
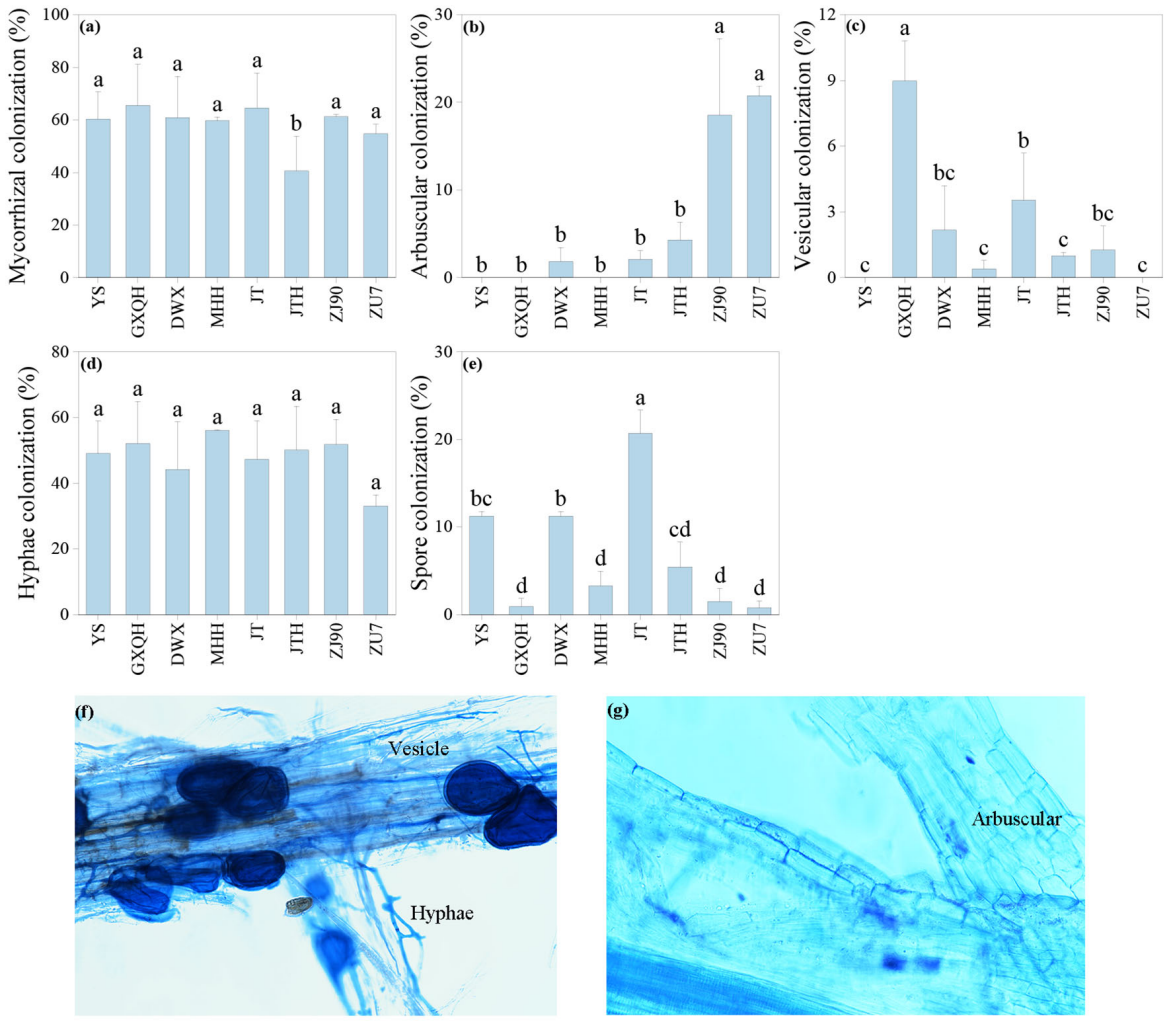
The AM colonization rate **(a)**, arbuscular **(b)**, vesicular **(c)**, hyphal **(d)**, and spore **(e)** colonization, along with hyphal, vesicle **(f)** and arbuscular **(g)** morphological structures in the rhizosphere of loquat. The figure included hypha and vesicle, is derived from ZJ90, while the arbuscular structure figure is sourced from ZU7. Different letters indicate significant differences (*P*<0.05). Error bars represent the mean value plus or minus the standard error (n=6). The same applies to the figures below.

All loquat varieties exhibit varying degrees of AMF colonization, with clear observation of structures such as spores, hyphae, and vesicles (**Figures 2a–g**). Analysis of infection rates revealed significant differences among different varieties (*P*<0.05). GXQH showed the highest overall colonization rate at 65.54%, contrasting with JTH, which displayed the lowest colonization rate of only 40.57% (**Figure 2a**). Regarding arbuscular colonization, ZU7 had the highest rate at 20.76%, followed by ZJ90 at 18.53%, while MHH, GXQH, and YS showed no arbuscular colonization (**Figure 2b**). Conversely, GXQH had the highest vesicular colonization rate at 9%, with YS and ZU7 observed no vesicles (**Figure 2c**). Notably, no significant difference was detected in mycelial colonization rates among the cultivars, with MHH showing the highest rate of 56.05% and ZU7 the lowest rate of 33.02% (**Figure 2d**). JT had the highest spore colonization rate of 20.69%, while ZU7 had the lowest rate of 0.77% (**Figure 2e**). Moreover, the density of AMF spores in the rhizosphere soil varied significantly across different loquat cultivars (**Table 1**). The root soil of YS exhibited the highest spore density at 64.02 spores/g, while ZU7 root soil had the lowest density at 12.57 spores/g. Spore diameters in JTH ranging from 226.01 μm to 294.44 μm, whereas those in JT had a smaller diameter range from 31.52 μm to 98.35 μm. The results suggest that different loquat cultivars vary significantly in mycorrhizal fungal infection rate, structural composition, abundance and size of AMF spores, which may affect their dependence on arbuscular mycorrhizal fungi and their interaction with soil.

**Table 1:** Comparison of AMF spore characters in the soils from eight loquat cultivars.

Species	Soil weight (g)	Spore count	Spore density	Spore diameter (μm)
YS	100.02	6403.67 ± 317.68a	64.02 ± 3.18a	85.20-125.10
GXQH	100.01	1612.67 ± 25.30ef	16.13 ± 0.25ef	93.31-157.26
DWX	100.02	2063.00 ± 75.61d	20.63 ± 0.76d	57.04-119.80
MHH	100.04	2332.00 ± 136.56d	23.31 ± 1.37d	115.07-128.16
JT	100.03	5019.67 ± 273.61b	50.18 ± 2.74b	31.52-98.35
JTH	100.01	1995.67 ± 237.06de	19.95 ± 2.37de	226.01-294.44
ZJ90	100.08	3890.67 ± 72.08c	38.88 ± 0.72c	52.02-93.21
ZU7	100.06	1258.00 ± 20.12f	12.57 ± 0.20f	44.63-101.65

Different letters represent different significance (*P*<0.05).

### 3.3 AMF diversity in the rhizosphere of different loquats

**Figure 3:**
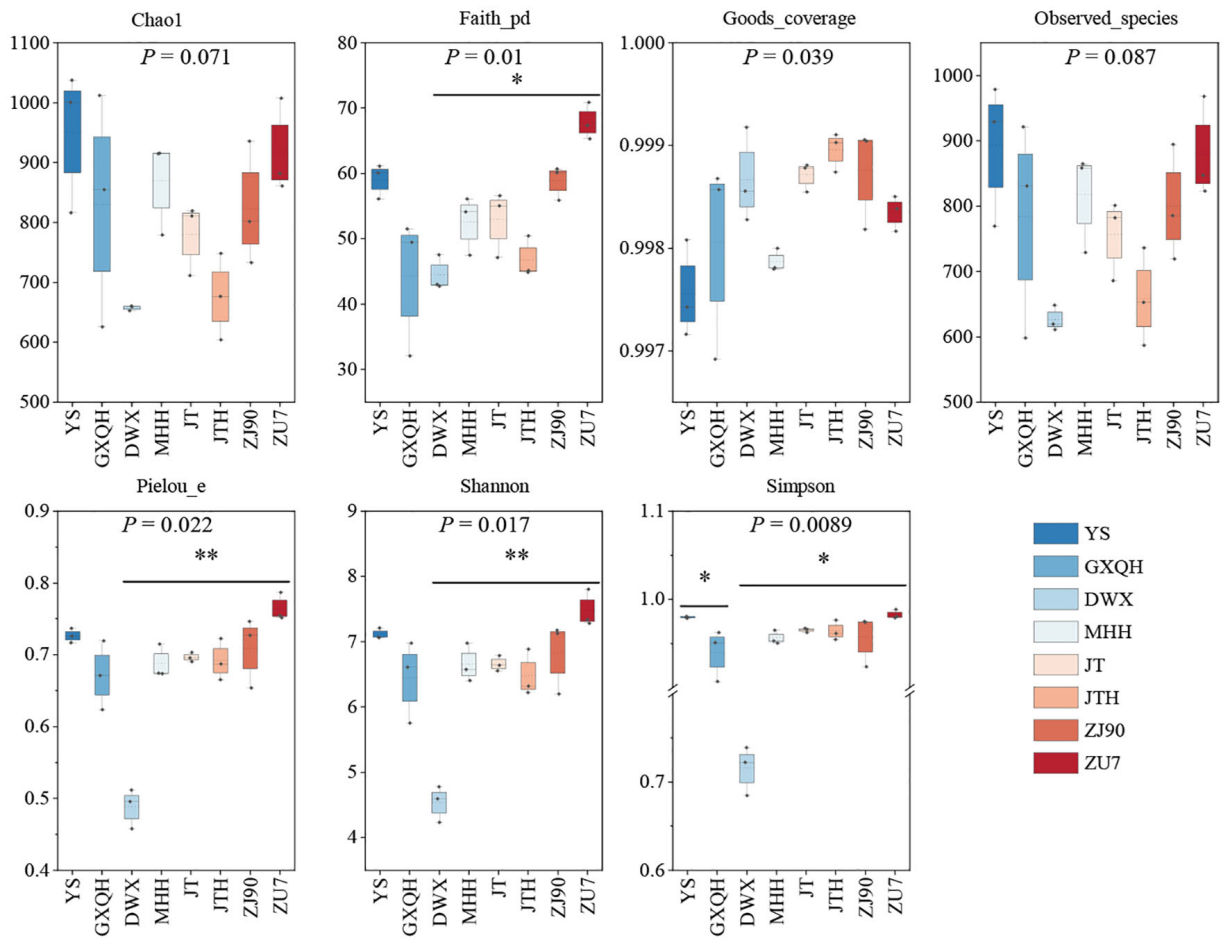
Alpha diversity index of soil AMF community in different varieties of loquat. Chao1 and Observed-species indices were used to characterize richness, Shannon and Simpson indices were used to characterize diversity, Faith’s PD index was used to characterize evolutionary diversity, Pielou’s evenness index was used to characterize evenness, and Good’s coverage index was used to characterize coverage. In the boxplot, the upper and lower lines of the box represent the upper and lower quartiles, the solid line represented the median, the dashed line represents the mean, and the upper and lower whiskers represented the maximum and minimum values. The numbers under the diversity index labels represented the *P*-values of the Kruskal-Wallis test. * indicated significance at *P* <0.05, ** indicated significance at *P* <0.01.

Analysis of AMF community composition (**Figure 3**) revealed that YS had higher Chao1 and Observed-species indices compared to other cultivars. However, the Shannon index, Simpson index, Faith-pd, Pielou-e, and Good-coverage indices showed no significant differences compared to other cultivars. Conversely, DWX displayed significantly lower Observed-species, Pielou-e, Shannon, and Simpson indices compared to other cultivars, suggesting low AMF richness and diversity in DWX. Similarly, JTH had lower Chao1 and Observed-species indices, as well as a lower Faith-pd index, suggesting reduced AMF community richness and evolutionary diversity compared to other cultivated cultivars. In contrast, ZU7 had higher levels across all indices except the Good-coverage index, indicating superior AMF community richness, fungal diversity, and community evenness compared to other cultivars. The richness of AMF associated with eight loquat plant roots, from highest to lowest, were YS, ZU7, MHH, GXQH, ZJ90, JT, JTH, and DWX.

**Figure 4:**
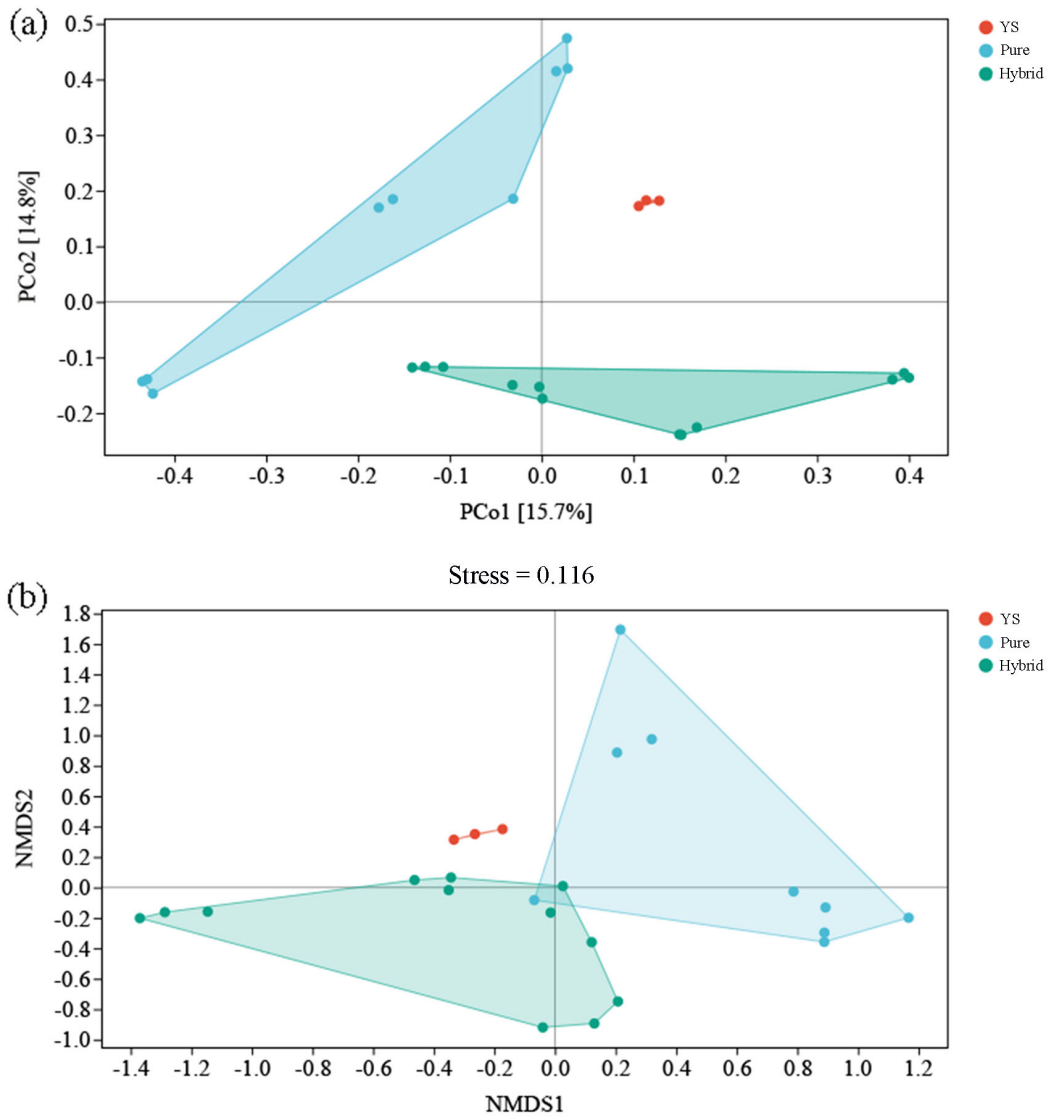
PCoA **(a)** and NMDS **(b)** analysis of AMF communities of different varieties loquat. Each point represents a sample, and points of different colors indicate different groups. PC1 represents the first principal component of AMF, and PC2 represents the second principal component.

Specifically, the PCoA (**Figure 4a**) and NMDS (**Figure 4b**) (stress=0.116) plots divided the AMF communities into three distinct clusters, pure species (MHH, GXQH, and DWX), hybrid species (JTH, JT, ZJ90, and ZU7), and wild species (YS). There were clear differences between the different varieties (**Figure 4**). In contrast, the first and second principal component scores of purebred varieties showed notable dispersion, indicating significant variation in the principal components among different varieties. Meanwhile, the first principal component scores of hybrid varieties were more concentrated, and the overall scores of wild varieties were also concentrated, suggesting that the main composition of AMF species in wild varieties is similar. The ANOSIM test (**Table 2**) using the Bray-Curtis distance metric provided further evidence that the composition of AMF communities varied significantly among different loquat cultivars (*P*=0.001). The results of PCoA and NMDS analyses were consistent, showing significant differences in the AMF communities of wild-type, purebred, and hybrid loquat varieties. The purebred communities were more like each other and distinctly different from the wild-type and hybrid-type communities.

**Table 2:** Analysis of differences in AMF communities among different loquat cultivars.

Microbial communities	PERMANOVA			Anosim			Permdisp		
	pseudo-F	*P*-value	q-value	pseudo-F	*P*-value	q-value	pseudo-F	*P*-value	q-value
YS/Pure	3.53	0.019^*^	0.019	-0.14	0.751	0.751	208.24	0.010^**^	0.018
YS/Hybrid	3.64	0.007^**^	0.010	0.23	0.077	0.1155	107.80	0.012^*^	0.018
Pure/Hybrid	3.73	0.001^**^	0.003	0.40	0.001^**^	0.003	0.120	0.621	0.621

PERMANOVA, Permutational multivariate analysis of variance; Anosim, Analysis of similarities; Permdisp, Permutation test of multivariate homogeneity of groups dispersions. * indicated significance at *P*<0.05, ** indicated significance at *P*<0.01.

### 3.4 Influence of loquat varieties on AMF composition

**Figure 5:**
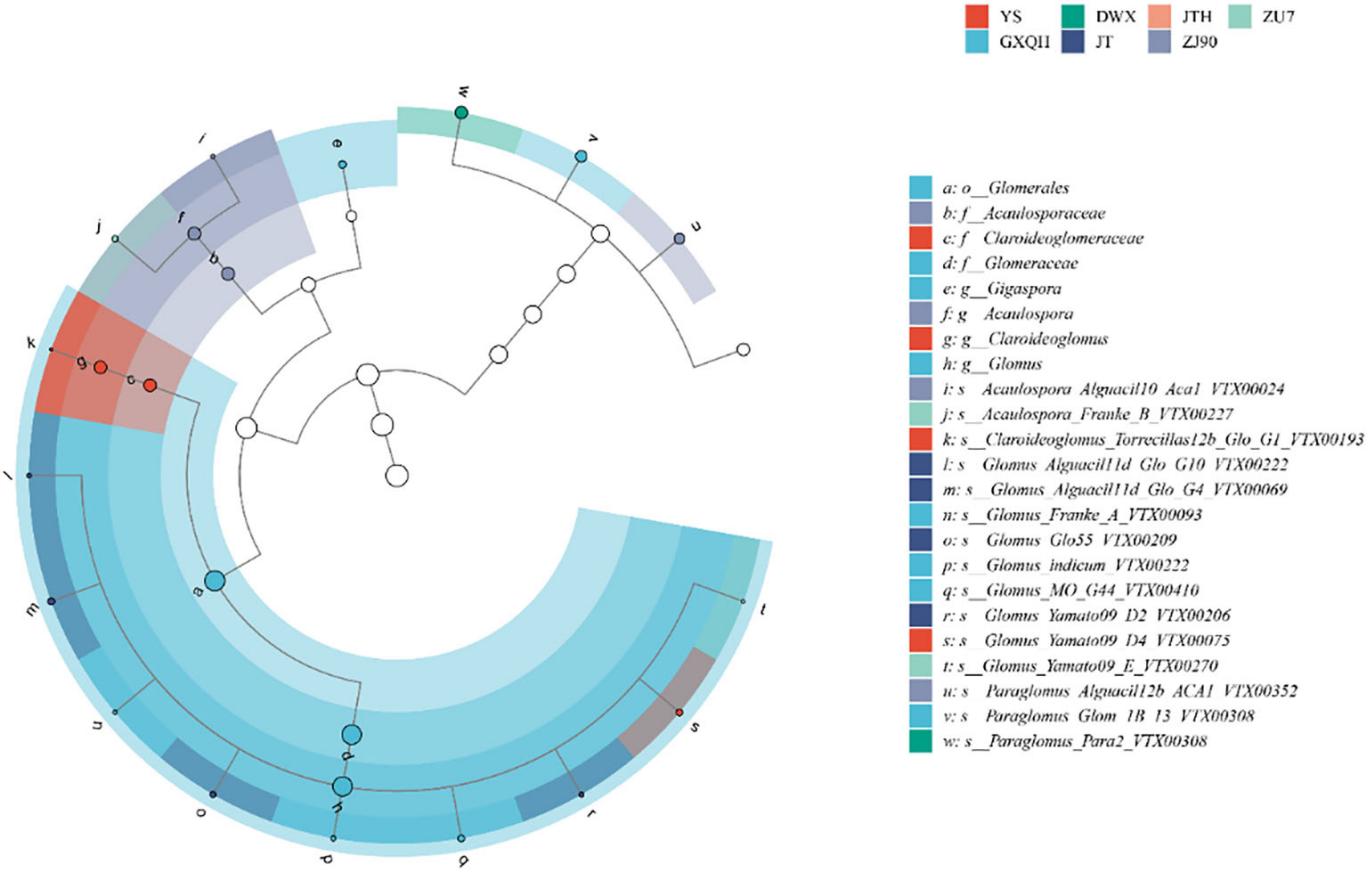
Lefse analysis based on the relative abundance of different ASVs within the AMF communities of each sample. The node size corresponds to the average relative abundance of each taxon. Hollow nodes represent taxa with no significant differences between groups, while colored nodes indicate taxa that show significant differences and have higher abundance in the corresponding groups. Letters denote the names of taxa with significant differences between groups.

The rhizosphere of loquat harbored diverse arbuscular mycorrhizal (AM) fungal communities across different cultivars, with a total of 999 OTUs/7231 ASVs detected. Distribution statistics of OTUs among various loquat varieties revealed that all loquat cultivars were associated with eight AMF genera. Eleven OTUs were shared among all eight loquat cultivars, belonging to *Glomus*, *Paraglomus*, and *Claroideoglomus*. ZU7 had the highest number of unique OTUs with 159, followed by MHH with 145, while DWX had the fewest unique OTUs with only 89 (**Supplementary Figure S2**). By classifying these eight species according to their ASVs, LEfSe analysis (**Figure 5**) showed clear differences that *Glomus* was most abundant (70.58%), followed by *Para*g*lomus* (11.49%).

**Figure 6:**
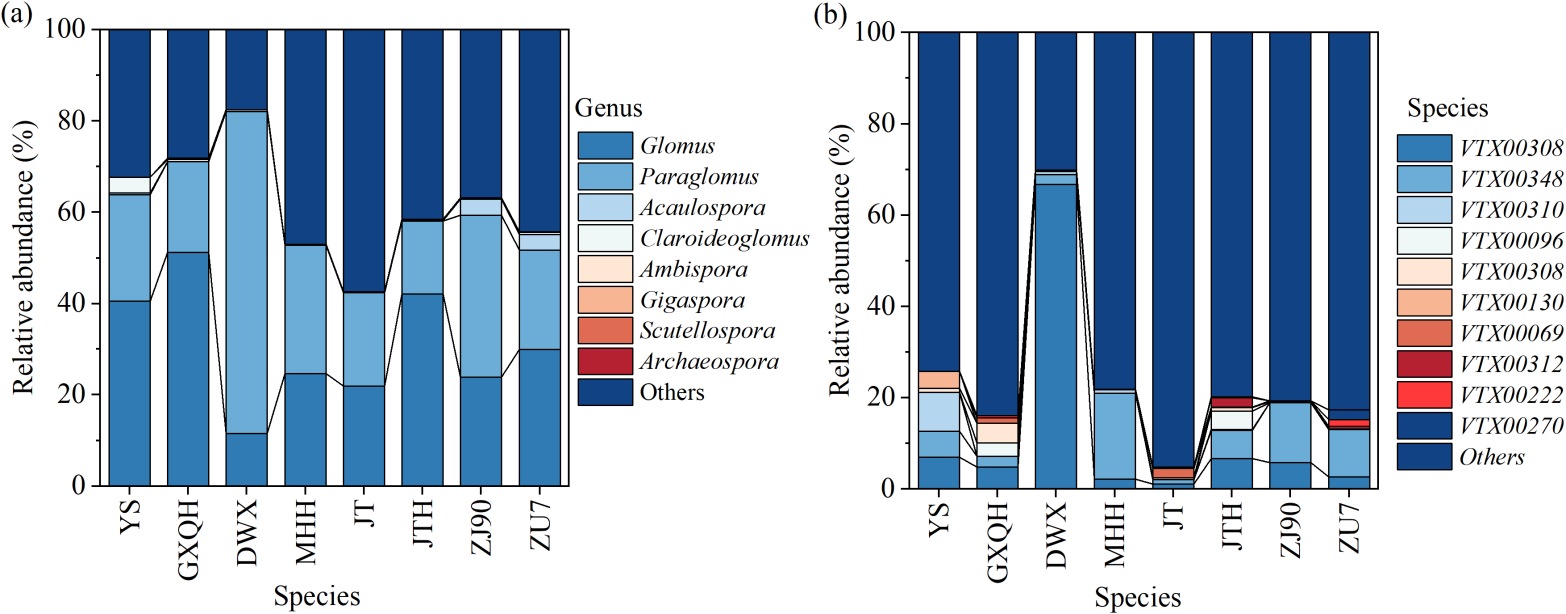
Relative abundance of key genus **(a)** and species **(b)** as effected by different loquat varieties.

The AMF community composition spanned 4 orders, 7 families, and 8 genera across the experimental soils. Dominant genera at the family level were Glomeraceae and Glomeraceae, comprising 30.72% and 29.46% of the total sequences, respectively. Further analysis at the genus level revealed *Glomus* (30.72%) and *Paraglomus* (29.46%) as the predominant genera (**Figure 6a**). In the rhizosphere of loquat, a total of 8 arbuscular mycorrhizal fungi genera were identified, including *Claroideoglomus*, *Glomus*, *Scutellospora*, *Gigaspora*, *Acaulospora*, *Archaeospora*, *Paraglomus*, and *Ambispora*. To ensure the accuracy of identification, spores isolated from the soil were morphologically identified based on the layers of spore wall and subtending hypha (**Supplementary Figure S1**). While *Glomus* and *Acaulospora* have been previously documented in association with loquat, the presence of other AMF genera represents novel findings. Notably, both *Glomus* and *Paraglomus* (VTX00308, VTX00348) exhibit a wide range of interactions with various loquat cultivars, being distributed across all varieties examined. However, there are significant differences in the distribution of AMF species among loquat populations (**Figure 6b**). For instance, GXQH and ZJ90 all had eight genera, with *Glomus* being most abundant in GXQH (51.26%). *Claroideoglomus* had the highest proportion in YS, accounting for 3.45%. *Ambispora* exclusively occurred in JT, while *Scutellospora* was confined to ZJ90 and *Gigaspora* solely inhabited GXQH. Despite the ubiquitous presence of *Glomus* in all loquat varieties, considerable diversity was observed at the species level (**Supplementary Table S2**). Specifically, *Glomus melanosporum* (VTX00069) and *Glomus lamellosum* were exclusive to GXQH and DWX, respectively, while *Glomus dolichoasporum* was restricted to DWX. Furthermore, *Acaulospora excavata* was specifically associated with MHH, whereas *Acaulospora foveata* was uniquely found in ZU7.

### 3.5 Correlations between AMF community and soil properties

**Figure 7:**
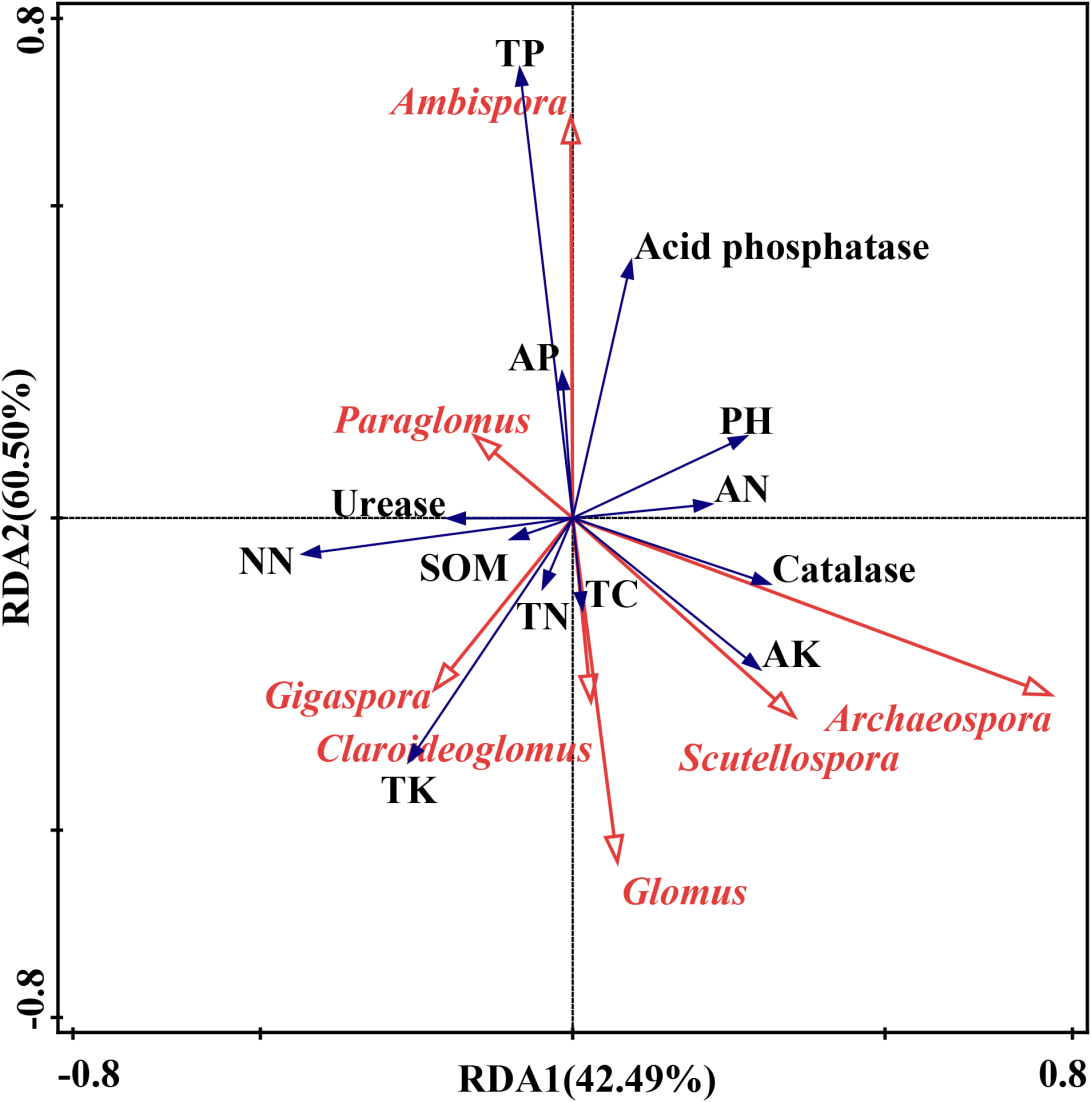
Redundancy analysis (RDA) between AMF genera (red arrow) and soil properties and enzyme activities (blue arrow). The axes explained 42.49% of the variance on RDA1 and 60.50% on RDA2. NN, nitrate nitrogen, AN, Ammonium Nitrogen. Other abbreviations were documented in **Figure 2** caption.

**Figure 8:**
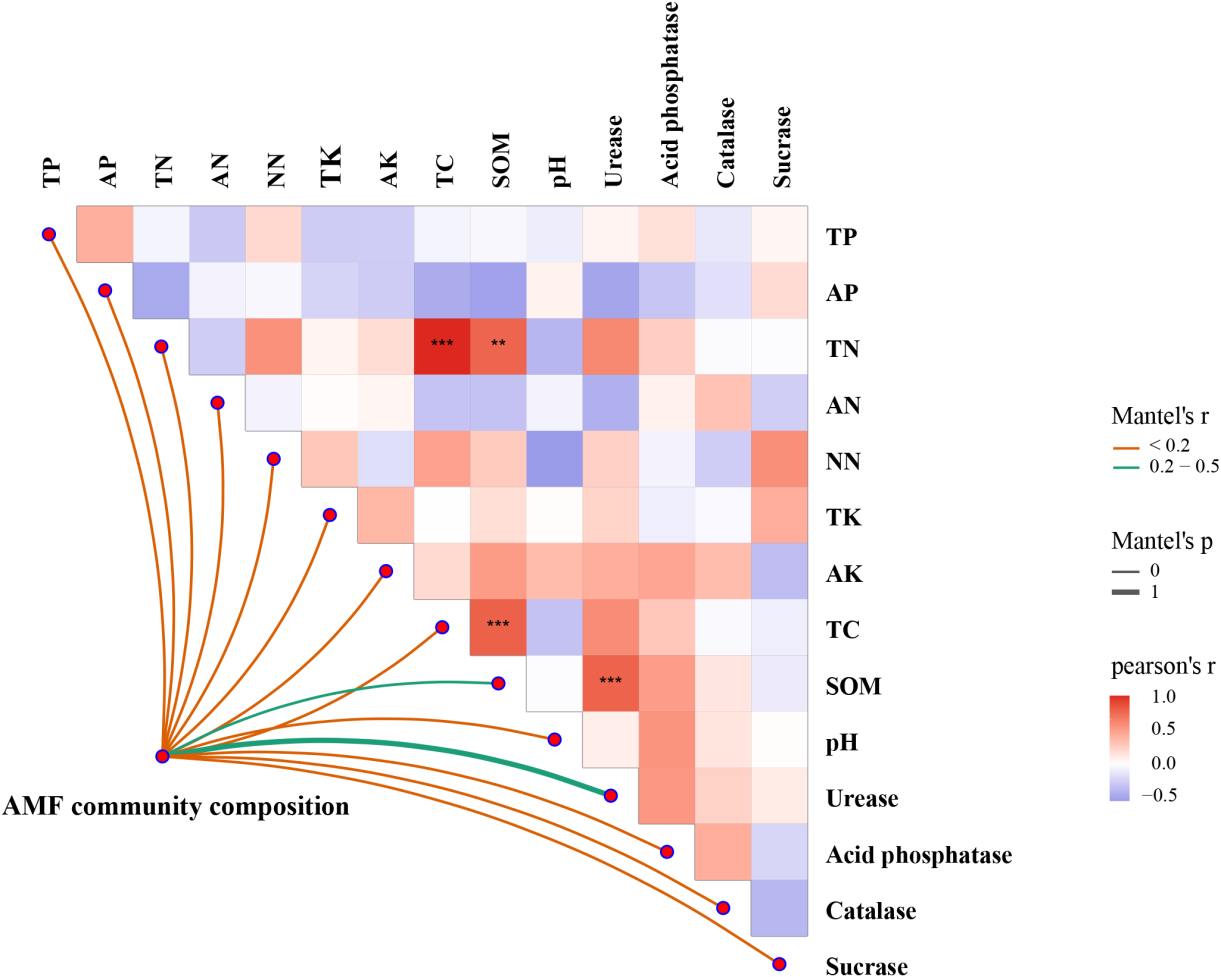
Relationships between AMF community composition and environmental factors based on Mantel tests. **Significant at *P*<0.01; ***Significant at *P*<0.001.

The RDA biplot demonstrated that different AMF genera were associated with distinct soil properties and enzyme activities (**Figure 7**). For instance, *Ambispora* was strongly associated with TP and acid phosphatase activity, suggesting a preference for phosphorus-rich environments, while *Glomus* tended to thrive in soils with lower potassium levels. *Paraglomus* correlated with urease activity and was linked to SOM and TN, indicating its presence in nutrient-rich soils. *Archaeospora*, *Scutellospora*, *Gigaspora* and *Claroideoglomus* were associated with potassium, suggesting their affinity for potassium. *Glomus*, however, was negatively correlated with AK, thriving in environments with low potassium availability. The results of the mantel test analysis indicated that TC (*P*<0.001) and SOM (*P*<0.001) were the primary drivers of AMF community composition, with strong, significant correlations (**Figure 8**). AK and TK showed moderate but less significant correlations. Other factors, including TN, total phosphorus TP, and acid phosphatase, had weak or nonsignificant correlations. pH also showed a weak, nonsignificant negative correlation with AMF composition. Overall, TC and SOM played the most critical roles in influencing AMF community structure.

### 3.6 The relationship between AMF composition and environmental factors

**Figure 9:**
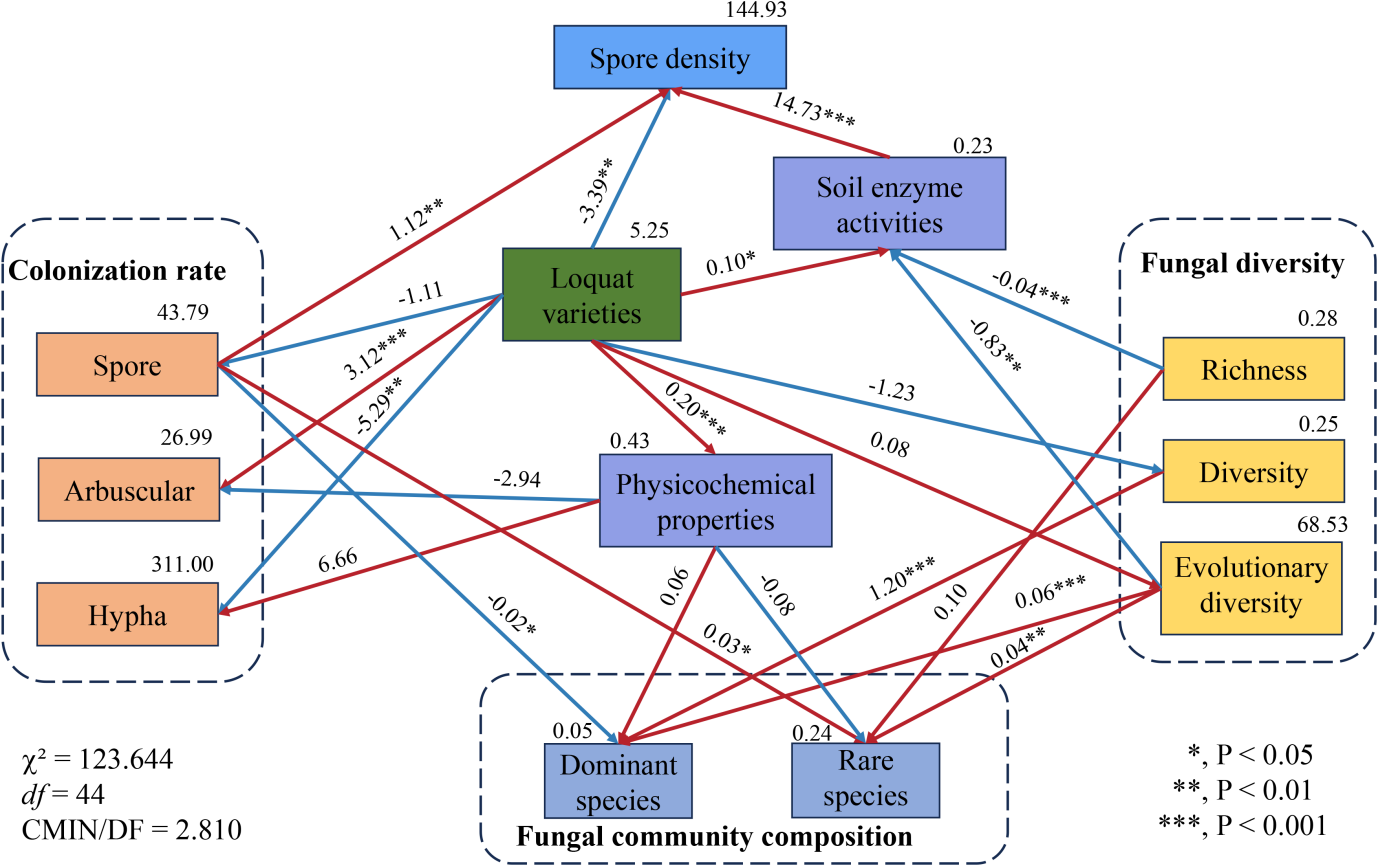
Structural equation model illustrating the causal relationships among loquat varieties, soil factors, and AMF parameters. The number above each arrow indicates the path coefficient. An asterisk (*) indicates a *P* value of less than 0.05, ** indicates *P*<0.01, and *** indicates *P*<0.001.

After analyzing the rhizosphere nutrient content and various AMF parameters (including mycorrhizal colonization, spore density, fungal diversity, and fungal community composition) of eight different loquat cultivars, we found significant variability among these factors, despite identical soil nutrient levels and consistent artificial nutrient inputs (**Figure 9**). To investigate this further, structural equation modeling (SEM) was used to explore the direct and indirect effects of loquat cultivars on AMF parameters. The SEM model was a good fit to the data, with a χ² value of 123.644 and a corresponding *P*-value of 0.000, indicating statistical significance. The CMIN/DF value of 2.810 suggests a moderate fit, with 44 degrees of freedom. Specifically, we observed direct and significant effects of cultivar on mycorrhizal colonization, spore density, and soil factors. Notably, cultivar had significant negative effects on mycelial colonization rate (−5.29, *P*<0.01) and spore density (−3.39, *P*<0.01), while it had significant positive effects on arbuscular colonization rate (3.12, *P*<0.001), soil physicochemical properties (0.20, *P*<0.001), and soil enzyme activity (0.10, *P*<0.05). This indicated that loquat cultivars play a key role in altering soil nutrients, regulating enzyme activity, and promoting arbuscular colonization of AM fungi. Interestingly, cultivar did not directly affect AMF diversity or community composition. However, mycorrhizal colonization had a significant negative impact on dominant AMF species (−0.02, *P*<0.05) and a significant positive impact on rare species (0.03, *P*<0.05). AMF diversity had a positive impact on dominant species (1.20, *P*<0.001). Notably, evolutionary diversity had a positive impact on both dominant species (0.06, *P*<0.001) and rare species (0.04, *P*<0.01), and had a direct negative impact on soil enzyme activity (−0.83, *P*<0.01).

## 4 Discussion

### 4.1 There are significant differences in the AMF in the rhizosphere of wild and cultivated loquat

The production of spores and hyphae, a part of the life cycle of AMF, is highly dependent on environmental factors (Ma et al., 2023). Our statistical analysis of colonization rates revealed varietal differences in AMF colonization forms, indicative of structural variations influenced by habitat alterations. As plants exhibit varying degrees of dependence on soil nutrients, corresponding allocation of AMF structures are observed (Ma et al., 2021). Given the diverse nutritional requirements of different loquat varieties, differences in AMF structures in the rhizosphere are expected. Notably, our findings highlighted *Glomus* as the dominant genus in GXQH, with its hyphae primarily contributing to root colonization, consistent with previous research (Hassan et al., 2011). The analysis of spore density revealed significant differences among cultivars, with overall spore density in the soil being higher than in root spore infections, indicating typical “soil-root” spatial heterogeneity. This may be due to limited root space and resources, which restrict the colonization of AMF in loquat roots, leading to lower abundance in the root system compared to the rhizosphere soil (Zhang et al., 2016). The rhizosphere soil of YS exhibited a higher spore density compared to both pure species and cultivated varieties. Interestingly, spore infection in the roots of pure species was greater than in wild species, maybe because wild species require more carbon sources for growth, which supports their overall development (Smith and Read, 2008; Cao et al., 2022).

### 4.2 The genetic traits of loquat varieties determine the differences in AMF community composition

In this study, we investigated the colonization of AMF in the rhizosphere of eight loquat varieties in the germplasm resource, revealing a consistent symbiotic relationship across all varieties. *Glomus* emerged as the most prevalent AMF genus, consistent with previous research highlighting its prevalence in loquat symbiosis (Xiao et al., 2023). Besides *Glomus*, our study found that AMF community was also dominated by *Paraglomus* and *Claroideoglomus* in all loquat varieties. Consistent with the findings of Guzman et al. (2021), *Claroideoglomus* had the largest proportion in intensive soils, alongside *Glomus*. However, different AMF exhibited specific selection for different cultivars to form a symbiotic relationship. For instance, *Scutellospora* was absent in YS, while *Gigaspora* and *Archaeospora* were not detected in DWX, JT, and JTH. These differences may result from long-term natural selection processes, as the association of AMF with plant hosts is usually non-random (Horn et al., 2017). Although AMF are present in each loquat variety, they display host selectivity, meaning that different loquat varieties may associate with different AMF species based on their specific growth requirements. Moreover, diversity indices indicated variations in AMF richness and community stability across different loquat varieties, with cultivated varieties boasting higher richness and more stable community structures. The higher organic matter content in the rhizosphere of the YS variety indicates that it can rely more on its own root system for nutrient absorption, reducing its dependence on AMF (Li et al., 2024). In contrast, the growth of cultivated varieties is more dependent on mycorrhizae. β-diversity analysis showed significant differences in AMF communities among wild, pure, and hybrid loquat varieties, with hybrid varieties being markedly different from both wild and pure varieties. These results highlight the influence of plant spatial structure on AMF distribution, reflecting the complex interactions between plant species and microbial communities (Bittebiere et al., 2020; Powell and Rillig, 2018). This suggests that AMF have adapted to the unique root environments of different loquat cultivars, showing an inherent selectivity for specific cultivars (Montesinos-Navarro et al., 2019).

### 4.3 AMF diversity decreased in nutrient-rich soils

In addition to aboveground plant species, soil factors are the main drivers for microbial community changes (Albracht et al., 2024; Zhang et al., 2024a). Among these, pH is an important predictor, particularly at finer spatial scales (Guzman et al., 2021). Previous research has shown that soil available phosphorus and latitude are the main factors affecting diversity, while available phosphorus and pH are the main influencing factors on abundance (Ma et al., 2023; Tao and Liu, 2024). Long-term fertilization increases soil nutrient leaching, forces soil acidification, and agricultural soils tend to select AMF populations with lower diversity and better adaptation to the environment (Thirkell et al., 2017). Our study identified soil pH as one of the environmental factors influencing the abundance of dominant AMF, with soil organic matter exhibiting a significant positive correlation with AMF community structure. This suggests that variations in soil pH and organic matter content influence the recruitment of different AMF communities in the roots, indirectly affecting the abundance of various AMF species (Baltruschat et al., 2019). Yang et al. (2024) found that the different distribution of arbuscular mycorrhizal fungi in soil affected the levels of glomalin-related soil proteins, thereby influencing the storage of organic carbon in the soil. In ecosystems with moderate competition, soils allocate more carbon and organic matter to AMF, compensating for the lack of AMF diversity (Konvalinková et al., 2017; Neuenkamp et al., 2021; Cheng et al., 2023). However, the addition of nutrients increases the biomass of the aboveground parts of the plant, reducing the restrictions on underground resources for the host plant (Liang et al., 2018; Ma et al., 2023). As a result, the carbon allocated by the host plant to AMF decreases, intensifying competition between species, leading to the loss of some AMF species, and promoting the dominance of certain AMF fungi (Bachelot and Lee, 2018; Zheng et al., 2018; Thirkell et al., 2020). Meng et al. (2024) demonstrated that while nitrogen fertilization significantly enhanced corn yield, it concurrently led to a notable reduction in the diversity of rhizosphere fungi. Structural equation models revealed that loquat varieties had a negative impact on AMF spore and hyphae colonization in the rhizosphere, as well as on spore density in the soil. Conversely, soil nutrients positively influenced *Glomus* and *Paraglomus* while negatively affecting other AMF species. These findings suggest that as planting duration increases, *Glomus* and *Paraglomus* are likely to become dominant across all loquat varieties, while *Ambispora* may decline due to competitive pressures.

## 5 Conclusions

In summary, this study demonstrated that different loquat varieties can be symbiosis with AMF, with infection rates ranging from 40.57% to 65.54%. High-throughput sequencing identified 15 AMF species across 8 genera in the loquat varieties, with *Glomus* and *Paraglomus* being the dominant genera. The results also showed that different varieties significantly affected soil nutrients and spores and hyphae of AM fungi, which in turn affected rare species of AMF, supporting the hypothesis that plant communities drive AMF composition. Additionally, the abundance and diversity of AMF in wild loquat species were higher than in cultivated and pure species. This effect is primarily attributed to the influence of TC and SOM. Long-term cultivation and fertilization practices can reduce AMF diversity and altered community composition, leading to the dominance of *Glomus* and *Paraglomus*. Furthermore, due to interspecific competition and nutrient enrichment, especially nitrogen and potassium, the population of *Ambispora* gradually declined. This study established the relationship between loquat varieties, AMF, and soil nutrients, providing insights to address challenges such as low yield and phosphorus deficiency in loquat cultivation. These results also have important implications for promoting sustainable environmental practices and mitigating soil acidification.

## Data Availability

The original contributions presented in the study are included in the article/**Supplementary Material**. Further inquiries can be directed to the corresponding author/s.

## References

[B1] Albracht C. Solbach M. Hennecke J. Bassi L. van der Ploeg G. Eisenhauer N.. (2024). Common soil history is more important than plant history for arbuscular mycorrhizal community assembly in an experimental grassland diversity gradient. Biol. Fertil. Soils. 60, 547–562. doi: 10.1007/s00374-024-01821-0

[B2] Bachelot B. Lee C. (2018). Dynamic preferential allocation to arbuscular mycorrhizal fungi explains fungal succession and coexistence. Ecology 99, 372–384. doi: 10.1002/ecy.2080 29121390

[B3] Baltruschat H. Santos V. M. da Silva D. Schellenberg I. Deubel A. Sieverding E.. (2019). Unexpectedly high diversity of arbuscular mycorrhizal fungi in fertile Chernozem croplands in Central Europe. Catena 182, 104135. doi: 10.1016/j.catena.2019.104135

[B4] Bao S. (2000). “Determination of plant macronutrients,” in Soil agrochemical Analysis (China Agriculture Press, Beijing), 264–270.

[B5] Bittebiere A. Vandenkoornhuyse P. Maluenda E. Gareil A. Dheilly A. Coudouel S.. (2020). Past spatial structure of plant communities determines arbuscular mycorrhizal fungal community assembly. J. Ecol. 108, 546–560. doi: 10.1111/1365-2745.13279

[B6] Brundrett M. Melville L. Peterson L. (1994). Practical methods in mycorrhiza research: based on a workshop organized in conjunction with the ninth North American Conference on mycorrhizae (Guelph: Mycologue Publications), 161.

[B7] Cao Y. Li N. Lin J. Zhang Y. Ma X. Wu P. (2022). Root system-rhizosphere soil-bulk soil interactions in different Chinese fir clones based on fungi community diversity change. Front. Ecol. Evol. 10. doi: 10.3389/fevo.2022.1028686

[B8] Chen Y. Fu W. Xiao H. Zhai Y. Luo Y. Wang Y.. (2023). A review on rhizosphere microbiota of tea plant (*Camellia sinensis* L.): Recent insights and future perspectives. J. Agric. Food Chem. 71, 19165–19188. doi: 10.1021/acs.jafc.3c02423 38019642

[B9] Chen Y. L. Xu Z. W. Xu T. L. Veresoglou S. D. Yang G. W. Chen B. D. (2017). Nitrogen deposition and precipitation induced phylogenetic clustering of arbuscular mycorrhizal fungal communities. Soil Biol. Biochem. 115, 233–242. doi: 10.1016/j.soilbio.2017.08.024

[B10] Cheng Y. Rutten G. Liu X. Ma M. Song Z. Maaroufi N. I.. (2023). Host plant height explains the effect of nitrogen enrichment on arbuscular mycorrhizal fungal communities. New Phytol. 240, 399–411. doi: 10.1111/nph.19140 37482960

[B11] Guan S. (1986). “Soil enzyme activity measurement,” in Soil enzymes and their research methods (China Agriculture Press, Beijing), 274–339.

[B12] Guzman A. Montes M. Hutchins L. de la Cerda G. Yang P. Kakouridis A.. (2021). Crop diversity enriches arbuscular mycorrhizal fungal communities in an intensive agricultural landscape. New Phytol. 231, 447–459. doi: 10.1111/nph.17306 33638170 PMC9292320

[B13] Hassan S. Boon E. St-Arnaud M. Hijri M. (2011). Molecular biodiversity of arbuscular mycorrhizal fungi in trace metal-polluted soils. Mol. Ecol. 20, 3469–3483. doi: 10.1111/j.1365-294X.2011.05142.x 21668808

[B14] He X. Y. Wu L. J. Wang W. X. Xie P. J. Chen Y. H. Wang F. (2020). Amygdalin - A pharmacological and toxicological review. J. Ethnopharmacol. 254, 112717. doi: 10.1016/j.jep.2020.112717 32114166

[B15] Horn S. Hempel S. Verbruggen E. Rillig M. C. Caruso T. (2017). Linking the community structure of arbuscular mycorrhizal fungi and plants: a story of interdependence? ISME. J. 11, 1400–1411. doi: 10.1038/ismej.2017.5 28244977 PMC5437357

[B16] Huang X. Wang H. Qu S. Luo W. Gao Z. (2021). Using artificial neural network in predicting the key fruit quality of loquat. Food Sci. Nutr. 9, 1780–1791. doi: 10.1002/fsn3.2166 33747488 PMC7958548

[B17] Jing D. Liu X. He Q. Dang J. Hu R. Xia Y.. (2023). Genome assembly of wild loquat (*Eriobotrya japonica*) and resequencing provide new insights into the genomic evolution and fruit domestication in loquat. Hortic. Res. 10, uhac265. doi: 10.1093/hr/uhac265 36778182 PMC9909508

[B18] Kajihara K. Egan C. Swift S. Wall C. Muir C. Hynson N. (2022). Core arbuscular mycorrhizal fungi are predicted by their high abundance-occupancy relationship while host-specific taxa are rare and geographically structured. New Phytol. 234, 1464–1476. doi: 10.1111/nph.18058 35218016

[B19] Keeney D. Nelson D. (1982). “Nitrogen-Inorganic forms in: Methods of Soil Analysis: Chemical and microbiological properties,” in Soil Science of America. Eds. Page A. Miller R. Keeney D. (Madison, Wisconsin: ASA-SSSA), 674–682.

[B20] Konvalinková T. Püschel D. Řezáčová V. Gryndlerová H. Jansa J. (2017). Carbon flow from plant to arbuscular mycorrhizal fungi is reduced under phosphorus fertilization. Plant Soil 419, 319–333. doi: 10.1007/s11104-017-3350-6

[B21] Li L. Shi Y. Xia W. Wang X. Xin Z. Liao Y.. (2024). Soil amendments altered arbuscular mycorrhizal fungal communities in cadmium-contaminated vegetable fields. Front. Microbiol. 15. doi: 10.3389/fmicb.2024.1470137 PMC11609156 39624713

[B22] Liang J. An J. Gao J. Zhang X. Yu F. (2018). Effects of arbuscular mycorrhizal fungi and soil nutrient addition on the growth of Phragmites australis under different drying-rewetting cycles. PloS One 13, e0191999. doi: 10.1371/journal.pone.0191999 29377943 PMC5788386

[B23] Liu Q. Parsons A. J. Xue H. Fraser K. Ryan G. D. Newman J. A.. (2011). Competition between foliar *Neotyphodium lolii* endophytes and mycorrhizal Glomus spp. fungi in *Lolium perenne* depends on resource supply and host carbohydrate content: Competitive endosymbiont interactions in *Lolium* . Funct. Ecol. 25, 910–920. doi: 10.1111/j.1365-2435.2011.01853.x

[B24] Luo Y. Wang Z. He Y. Li G. Lv X. Zhuang L. (2020). High-throughput sequencing analysis of the rhizosphere arbuscular mycorrhizal fungi (AMF) community composition associated with *Ferula sinkiangensis* . BMC Microbiol. 20, 335. doi: 10.1186/s12866-020-02024-x 33143657 PMC7640387

[B25] Ma X. Geng Q. Zhang H. Bian C. Chen H. Y. H. Jiang D.. (2021). Global negative effects of nutrient enrichment on arbuscular mycorrhizal fungi, plant diversity and ecosystem multifunctionality. New Phytol. 229, 2957–2969. doi: 10.1111/nph.17077 33188641

[B26] Ma X. Xu X. Geng Q. Luo Y. Ju C. Li Q.. (2023). Global arbuscular mycorrhizal fungal diversity and abundance decreases with soil available phosphorus. Glob. Ecol. Biogeogr. 32, 1423–1434. doi: 10.1111/geb.13704

[B27] Maillet F. Poinsot V. André O. Puech-Pagès V. Haouy A. Gueunier M.. (2011). Fungal lipochitooligosaccharide symbiotic signals in arbuscular mycorrhiza. Nature 469, 58–63. doi: 10.1038/nature09622 21209659

[B28] McGonigle T. P. Miller M. H. Evans D. G. Fairchild G. L. Swan J. A. (1990). A new method which gives an objective measure of colonization of roots by vesicular-arbuscular mycorrhizal fungi. New Phytol. 115, 495–501. doi: 10.1111/j.1469-8137.1990.tb00476.x 33874272

[B29] Meng T. Shi J. Zhang X. Zhao X. Zhang D. Chen L.. (2024). Slow-release nitrogen fertilizer application regulated rhizosphere microbial diversity to increase maize yield. Front. Plant Sci. 15. doi: 10.3389/fpls.2024.1481465 PMC11620899 39649805

[B30] Mhlanga B. Ercoli L. Piazza G. Thierfelder C. Pellegrino E. (2022). Occurrence and diversity of arbuscular mycorrhizal fungi colonising off-season and in-season weeds and their relationship with maize yield under conservation agriculture. Biol. Fertil. Soils. 58, 917–935. doi: 10.1007/s00374-022-01678-1

[B31] Montesinos-Navarro A. Valiente-Banuet A. Verdú M. (2019). Mycorrhizal symbiosis increases the benefits of plant facilitative interactions. Ecogr. (Cop.). 42, 447–455. doi: 10.1111/ecog.03926

[B32] Neuenkamp L. Zobel M. Koorem K. Jairus T. Davison J. Öpik M.. (2021). Light availability and light demand of plants shape the arbuscular mycorrhizal fungal communities in their roots. Ecol. Lett. 24, 426–437. doi: 10.1111/ele.13656 33319429

[B33] Olsen S. Sommers L. (1982). “Phosphorous,” in Methods of Soil Analysis, Part 2, Chemical and Microbial Properties. Eds. Page A. Miller R. Keeney D. (Agronomy Society of America, Agronomy Monograph 9, Madison, Wisconsin), 403–430.

[B34] Phillips J. Hayman D. (1970). Improved procedures for clearing roots and staining parasitic and vesicular-arbuscular mycorrhizal fungi for rapid assessment of infection. Trans. Br. Mycol. Soc 55, 158–161. doi: 10.1016/s0007-1536(70)80110-3

[B35] Polat A. Durgac C. Caliskan O. (2005). Effect of protected cultivation on the precocity, yield and fruit quality in loquat. Sci. Hortic. (Amsterdam). 104, 189–198. doi: 10.1016/j.scienta.2004.08.010

[B36] Powell J. R. Rillig M. C. (2018). Biodiversity of arbuscular mycorrhizal fungi and ecosystem function. New Phytol. 220, 1059–1075. doi: 10.1111/nph.15119 29603232

[B37] Sha Z. Watanabe T. Chu Q. Oka N. Osaki M. Shinano T. (2019). A reduced phosphorus application rate using a mycorrhizal plant as the preceding crop maintains soybean seeds’ nutritional quality. J. Agric. Food Chem. 67, 32–42. doi: 10.1021/acs.jafc.8b05288 30525606

[B38] Sheldrake M. Rosenstock N. P. Mangan S. Revillini D. Sayer E. J. Olsson P. A.. (2018). Responses of arbuscular mycorrhizal fungi to long-term inorganic and organic nutrient addition in a lowland tropical forest. ISME. J. 12, 2433–2445. doi: 10.1038/s41396-018-0189-7 29899509 PMC6155082

[B39] Smith S. Read D. (2008). “Colonization of roots and anatomy of arbuscular mycorrhizas,” in Mycorrhizal symbiosis (Academic Press, New York, New York State), 42–90.

[B40] Tao J. Liu X. (2024). Does arbuscular mycorrhizal fungi inoculation influence soil carbon sequestration? Biol. Fertil. Soils. 60, 213–225. doi: 10.1007/s00374-024-01793-1

[B41] Thirkell T. Charters M. Elliott A. Sait S. Field K. (2017). Are mycorrhizal fungi our sustainable saviours? Considerations for achieving food security. J. Ecol. 105, 921–929. doi: 10.1111/1365-2745.12788

[B42] Thirkell T. Pastok D. Field K. (2020). Carbon for nutrient exchange between arbuscular mycorrhizal fungi and wheat varies according to cultivar and changes in atmospheric carbon dioxide concentration. Glob. Change Biol. 26, 1725–1738. doi: 10.1111/gcb.14851 PMC7079082 31645088

[B43] Wahab A. Muhammad M. Munir A. Abdi G. Zaman W. Ayaz A.. (2023). Role of arbuscular mycorrhizal fungi in regulating growth, enhancing productivity, and potentially influencing ecosystems under abiotic and biotic stresses. Plants 12 (17), 3102. doi: 10.3390/plants12173102 37687353 PMC10489935

[B44] Wang F. Rengel Z. (2024). Disentangling the contributions of arbuscular mycorrhizal fungi to soil multifunctionality. Pedosphere 34, 269–278. doi: 10.1016/j.pedsph.2023.12.015

[B45] Wang J. Gao X. Wang J. Song J. Zhu Z. Zhao J.. (2024). Host plants directly determine the α diversity of rhizosphere arbuscular mycorrhizal fungal communities in the National Tropical Fruit Tree Field Genebank. Chem. Biol. Technol. Agric. 11, 20. doi: 10.1186/s40538-024-00540-w

[B46] Wang X. L. Yao Q. Feng Q. R. Huang J. L. Hu Y. L. (2007). Morphological characteristics of loquat mycorrhiza and inoculation effects of arbuscular mycorrhizal fungi on loquat. Acta Hortic. 750, 389–394. doi: 10.17660/actahortic.2007.750.62

[B47] Wen Z. Yang M. Fazal A. Han H. Lin H. Yin T.. (2024). Harnessing the power of microbes: Enhancing soybean growth in an acidic soil through AMF inoculation rather than P-fertilization. Hortic. Res. 11, uhae067. doi: 10.1093/hr/uhae067 38725460 PMC11079484

[B48] Wu Y. Chen C. Wang G. (2024b). Inoculation with arbuscular mycorrhizal fungi improves plant biomass and nitrogen and phosphorus nutrients: a meta-analysis. BMC Plant Biol. 24, 960. doi: 10.1186/s12870-024-05638-9 39396962 PMC11472555

[B49] Wu X. Ma C. Jiang H. Zhang X. Wang H. Li H.. (2024a). Root endophyte-manipulated alteration in rhizodeposits stimulates *Claroideoglomus* in the rhizosphere to enhance drought resistance in peanut. J. Agric. Food Chem. 72, 20211–20223. doi: 10.1021/acs.jafc.4c05009 39197047

[B50] Xiang X. De K. Lin W. Feng T. Li F. Wei X. (2024). Indirect influence of soil enzymes and their stoichiometry on soil organic carbon response to warming and nitrogen deposition in the Tibetan *Plateau alpine* meadow. Front. Microbiol. 15. doi: 10.3389/fmicb.2024.1381891 PMC11061507 38694804

[B51] Xiao D. Gai S. He X. Zhang W. Hu P. Soromotin A. V.. (2023). Habitat heterogeneity drives arbuscular mycorrhizal fungi and shrub communities in karst ecosystems. Catena 233, 107513. doi: 10.1016/j.catena.2023.107513

[B52] Xu F. Chu C. Xu Z. (2020). Effects of different fertilizer formulas on the growth of loquat rootstocks and stem lignification. Sci. Rep. 10, 1033. doi: 10.1038/s41598-019-57270-5 31974494 PMC6978351

[B53] Yang H. Wang G. Wang J. Xiao Q. Li Z. De Clerck C.. (2024). No-tillage facilitates soil organic carbon sequestration by enhancing arbuscular mycorrhizal fungi-related soil proteins accumulation and aggregation. Catena 245, 108323. doi: 10.1016/j.catena.2024.108323

[B54] Zhang M. Shi Z. Wang F. (2024b). Co-occurring tree species drive arbuscular mycorrhizal fungi diversity in tropical forest. Int. Microbiol. 27, 917–928. doi: 10.1007/s10123-023-00443-0 37923942

[B55] Zhang C. Xiang X. Yang T. Liu X. Ma Y. Zhang K.. (2024a). Nitrogen fertilization reduces plant diversity by changing the diversity and stability of arbuscular mycorrhizal fungal community in a temperate steppe. Sci. Total. Environ. 918, 170775. doi: 10.1016/j.scitotenv.2024.170775 38331277

[B56] Zhang Y. Yao Q. Li J. Hu Y. Chen J. (2014). Growth response and nutrient uptake of Eriobotrya japonica plants inoculated with three isolates of arbuscular mycorrhizal fungi under water stress condition. J. Plant Nutr. 37, 690–703. doi: 10.1080/01904167.2013.868478

[B57] Zhang D. Zhang C. Tang X. Li H. Zhang F. Rengel Z.. (2016). Increased soil phosphorus availability induced by faba bean root exudation stimulates root growth and phosphorus uptake in neighbouring maize. New Phytol. 209, 823–831. doi: 10.1111/nph.13613 26313736

[B58] Zheng Z. Ma P. Li J. Ren L. Bai W. Tian Q.. (2018). Arbuscular mycorrhizal fungal communities associated with two dominant species differ in their responses to long-term nitrogen addition in temperate grasslands. Funct. Ecol. 32, 1575–1588. doi: 10.1111/1365-2435.13081

[B59] Zhu B. Gao T. Zhang D. Ding K. Li C. Ma F. (2022). Functions of arbuscular mycorrhizal fungi in horticultural crops. Sci. Hortic. (Amsterdam). 303, 111219. doi: 10.1016/j.scienta.2022.111219

