# Supplementary material for: Impact of long-term loquat cultivation on rhizosphere soil characteristics and AMF community structure: implications for fertilizer management
Source: Front Plant Sci. 2025 Mar 13;16:1549384. doi: 10.3389/fpls.2025.1549384 (PMC11966047; doi:10.3389/fpls.2025.1549384)
Supplement: Supplementary file 1 [file DataSheet1.docx]

**Supplementary Information**

**
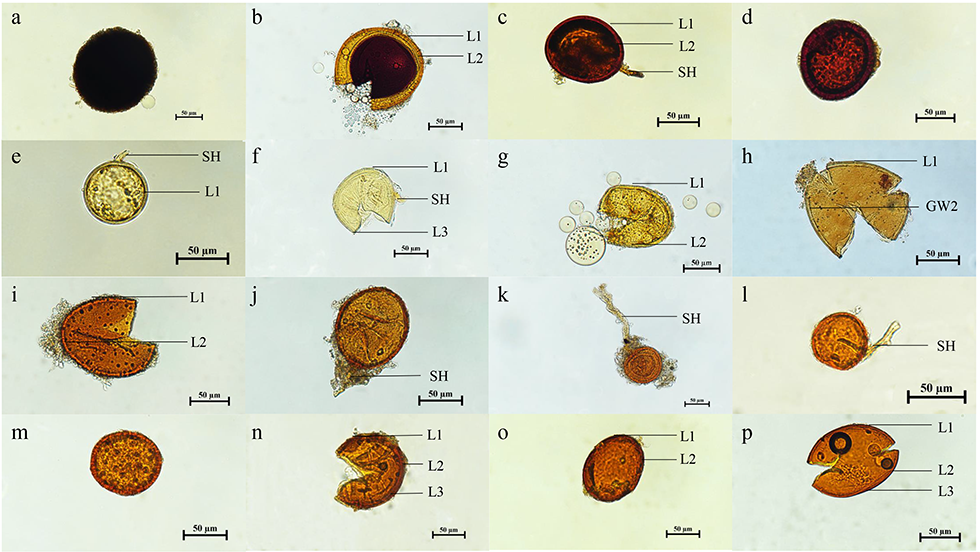
**

**Fig. S1** Morphological identification of AMF in eight loquat varieties. L is layers of spore wall; SH is subtending hypha. The figures are arranged in the order in Table S3.

**
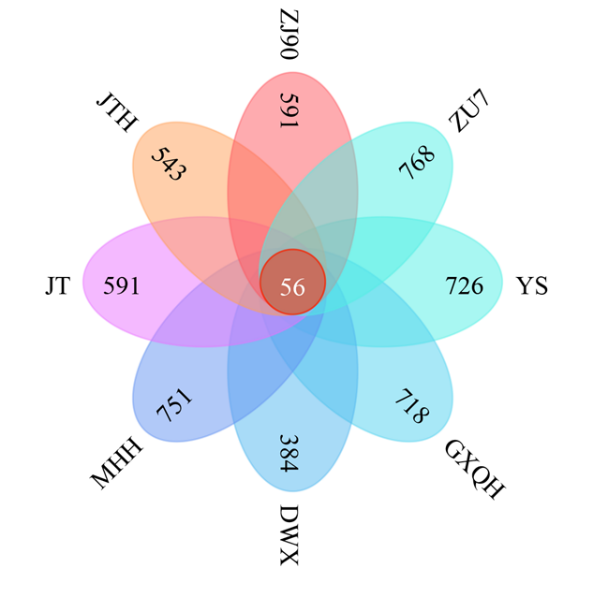
**

**Fig. S2** OTU/ASVs composition of AMF in eight loquat varieties. Each color block represents a group, and the overlapping area between blocks indicates the ASV/OTU shared by the corresponding groups. The number in each block indicates the number of ASV/OTU included in that block.

**Table S1.** Sample information and sampling site of different varieties of loquat

| Abbreviation | Species | Hybrid | Altitude | Longitude and latitude |
| --- | --- | --- | --- | --- |
| YS | Wild Type | / | 35.15 ± 3.3 | E113^°^21′21″  N23°9′35″ |
| GXQH | Guangxi QiuHua | / | 33.53 ± 4.9 | E113°21′13″  N23°9′79″ |
| DWX | DaWuXing | / | 31.00 ± 3.3 | E113°21′22″  N23°9′27″ |
| MHH | MaiHouHuang | / | 42.58 ± 4.9 | E113°21′41″  N23°9′27″ |
| JT | JieTuo | Jiefangzhong × *Eriobotrya elliptica* | 39.50 ± 3.0 | E113°21′12″  N23°9′82″ |
| JTH | JieTaiHeng | Zaozhong 6 × *Eriobotrya deflexa* (Hemsl.) Nakai form. *koshunensis* (Kaneh. & Sasaki) H. L. L. | 43.05 ± 3.0 | E113°21′13″  N23°9′82″ |
| ZJ90 | ZaoJia 90 | Zaozhong 6 × Jaierin | 39.50 ± 3.0 | E113°21′11″  N23°9′81″ |
| ZU7 | Yuehui 7 | Zaozhong 6 × Ullera | 40.96 ± 3.0 | E113°21′11″  N23°9′77″ |

Note: The altitudes, longitude and latitude listed in the table were measured using the Aowei Interactive Map (Beijing Yuansheng Huawang Software Co., Ltd, Beijing, China).

**Table S2.** Identification of AMF and their distribution in loquat

| AM fungal genera | AM fungal species | ASV/OTU | Detected in the different loquat varieties |
| --- | --- | --- | --- |
| *Claroideoglomus* | *Claroideoglomus claroideum* | VTX00055, VTX00057, VTX00064, VTX00185, VTX00193, VTX00214, VTX00276, VTX00278, VTX00297, VTX00316, VTX00317, VTX00340, VTX00341, VTX00357, VTX00358, VTX00402, VTX000427, VTX00441 | YS, GXQH, DWX, ZU7 |
| *Glomus* | *Glomus melanosporum* | VTX00069, VTX00076, VTX00077, VTX00085, VTX00088, VTX00121, VTX00122, VTX00124, VTX00126 VTX00132, VTX00140, VTX00143, VTX00151, VTX00174, VTX00179, VTX000181, VTX00183, VTX00189, VTX00194, VTX00206, VTX00209, VTX00216, VTX00223, VTX00270, VTX00288, VTX00366, VTX00367, VTX00370, VTX00371, VTX00372, VTX00373, VTX00383, VTX00407, VTX00410, VTX00418, VTX00419, VTX00422, VTX00423, VTX00437, VTX00443, VTX00448, VTX00453 | GXQH, DWX |
|  | *Glomus* *lamellosum* |  | GXQH, DWX |
|  | *Glomus* *fasciculatum* |  | YS, ZJ90 |
|  | *Glomus multiforum* |  | MHH, ZU7 |
|  | *Glomus delhiense* |  | YS, JT, JTH |
|  | *Glomus dolichoasporum* |  | DWX |
| *Scutellospora* | *Scutellospora* sp*.* | VTX00052, VTX00199, VTX00254, VTX00260 | ZJ90 |
| *Gigaspora* | *Gigaspora margarita* | VTX00039 | JT, ZJ90 |
| *Acaulospora* | *Acaulospora excavate* | VTX00010, VTX00047, VTX00227, VTX00249, VTX00351, VTX00352, VTX00379 | MHH |
|  | *Acaulospora laevis* |  | MHH, ZJ90 |
|  | *Acaulospora foveate* |  | DWX |
| *Archaeospora* | *Archaeospora* sp*.* | VTX00004, VTX00051, VTX000245, VTX00338, VTX00376, VTX000450, VTX00456 | GXQH, DWX, MHH |
| *Paraglomus* | *Paraglomus* sp*.* | VTX00238, VTX00239, VTX00281, VTX00308, VTX00337, VTX00348, VTX00350, VTX00375, VTX00433, VTX00435, VTX00444, VTX00446 | YS, GXQH, DWX, MHH, JT, JTH, ZJ90, ZU7 |
| *Ambispora* | *Ambispora* sp*.* | VTX00008, VTX00242, VTX00283, VTX00405 | JT |
